# Supplementary material for: 790,000 years of millennial-scale Cape Horn Current variability and interhemispheric linkages
Source: Nat Commun. 2025 Apr 1;16:3105. doi: 10.1038/s41467-025-58458-2 (PMC11958772; doi:10.1038/s41467-025-58458-2)
Supplement: Supplementary file 1 — Supplementary Information [file 41467_2025_58458_MOESM1_ESM.pdf]

Supporting information for "790,000 years of millennial-scale Cape Horn Current variability and interhemispheric linkages"

V. Rigalleau*, F. Lamy, N. Ruggieri, H. Sadatzki, H.W. Arz, S. Barker, L. Lembke-Jene, A. Wegwerth, G. Knorr, I.M. Venancio, T.M. L. Pinho, R. Tiedemann & G. Winckler

*Corresponding author. Email: vincent.rigalleau@awi.de

This PDF file includes Supplementary figures 1 to 12f

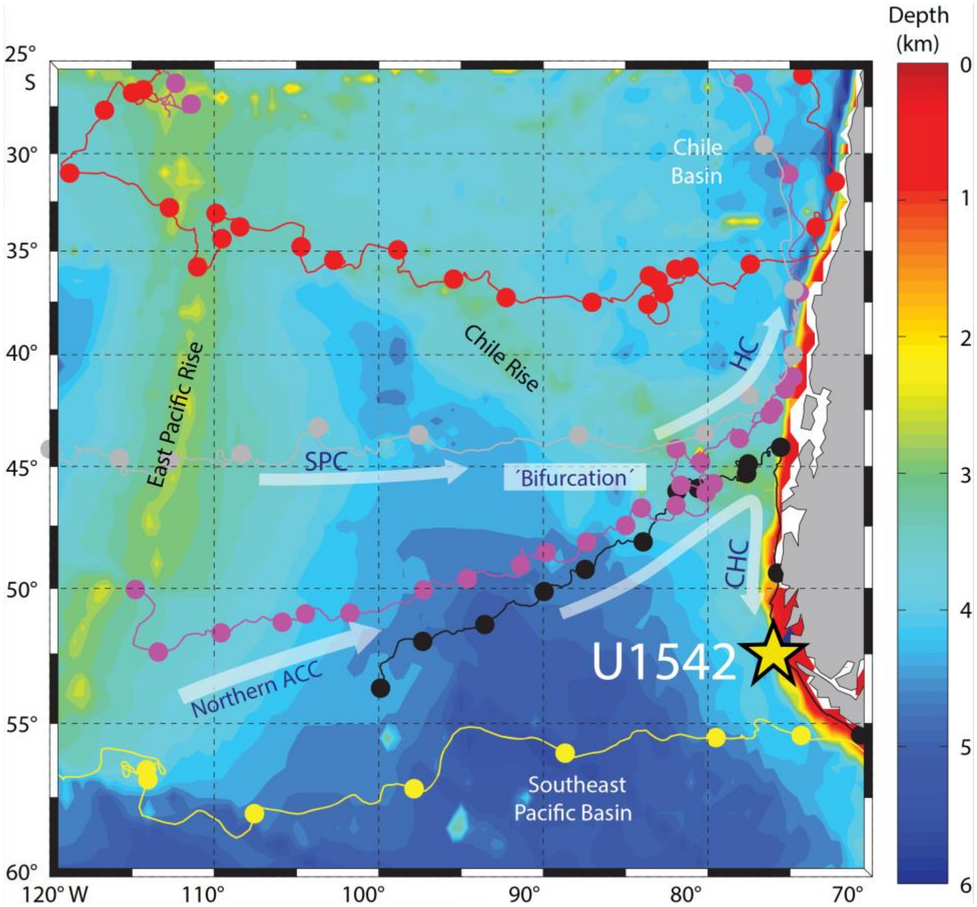

Supplementary figure 1. Surface circulation in the Eastern South Pacific with examples of surface buoy trajectories (each 30-day position is marked by a circle) indicating eastward flow of the South Pacific Current (SPC) and the northern Antarctic Circumpolar Current (ACC) water after crossing the East Pacific Rise. When the SPC impinges the Chilean coast, it bifurcates (at ~44°S) with northward flowing water in the Humboldt Current (HC) (represented by the grey and purple buoy[1]) and southward flow in the Cap Horn Current (CHC) toward the Drake Passage[2] (black drifter). After being transported via the CHC, the black "drifter" returns to the ACC two months later[1]. The CHC is described as a component that acts as an inter-basin conduit, drawing relatively cold and fresh Subantarctic Surface Water from the South Pacific, and injecting it into the South Atlantic. Recent remote sensing studies[2–4] show that the geostrophic CHC intensifies south of 49°S, with transport increasing from 0.4 Sv near 49°S to 5.3 Sv at Cape Horn, where it finally merges with the Sub-Antarctic Front of the ACC system, and represents 10% of the Drake Passage throughflow. This implies that the CHC can be considered a component of the overall ACC system and can be characterized as a northern, subantarctic branch of the ACC[3]. Modified from the original physical oceanography study by Chaigneau and Pizarro[1] and the paleoceanography study by Lamy et al.[5].

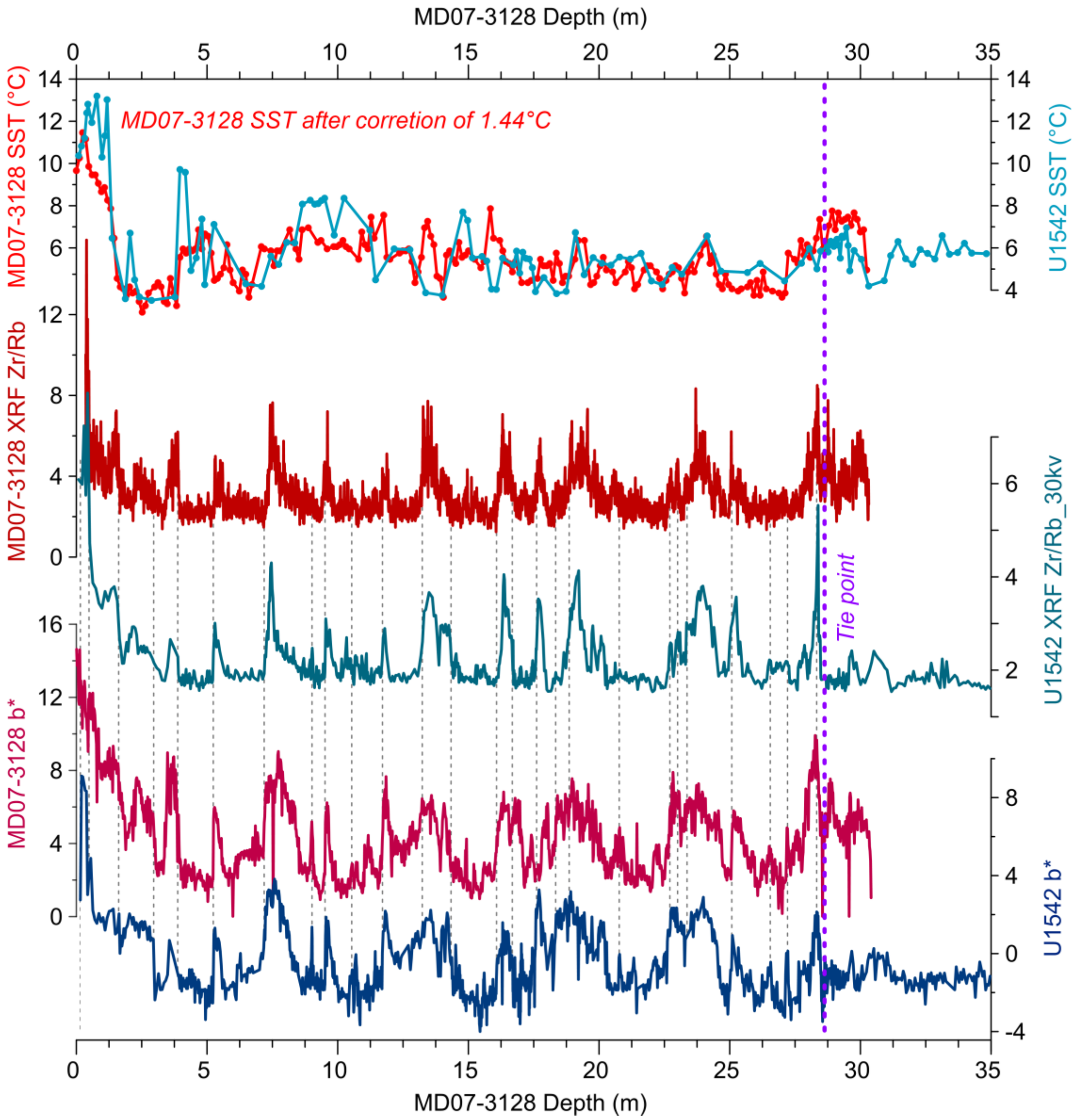

Supplementary figure 2. The appending of U1542 (blue) to the bottom of MD07-3128 (red). SST, X-Ray Fluorescence (XRF) Zr/Rb, and b* are the main proxies used to correlate the two records. b* was mainly used to correlate both records with control of XRF Zr/Rb ratio. All tuning was performed using AnalySeries[6].

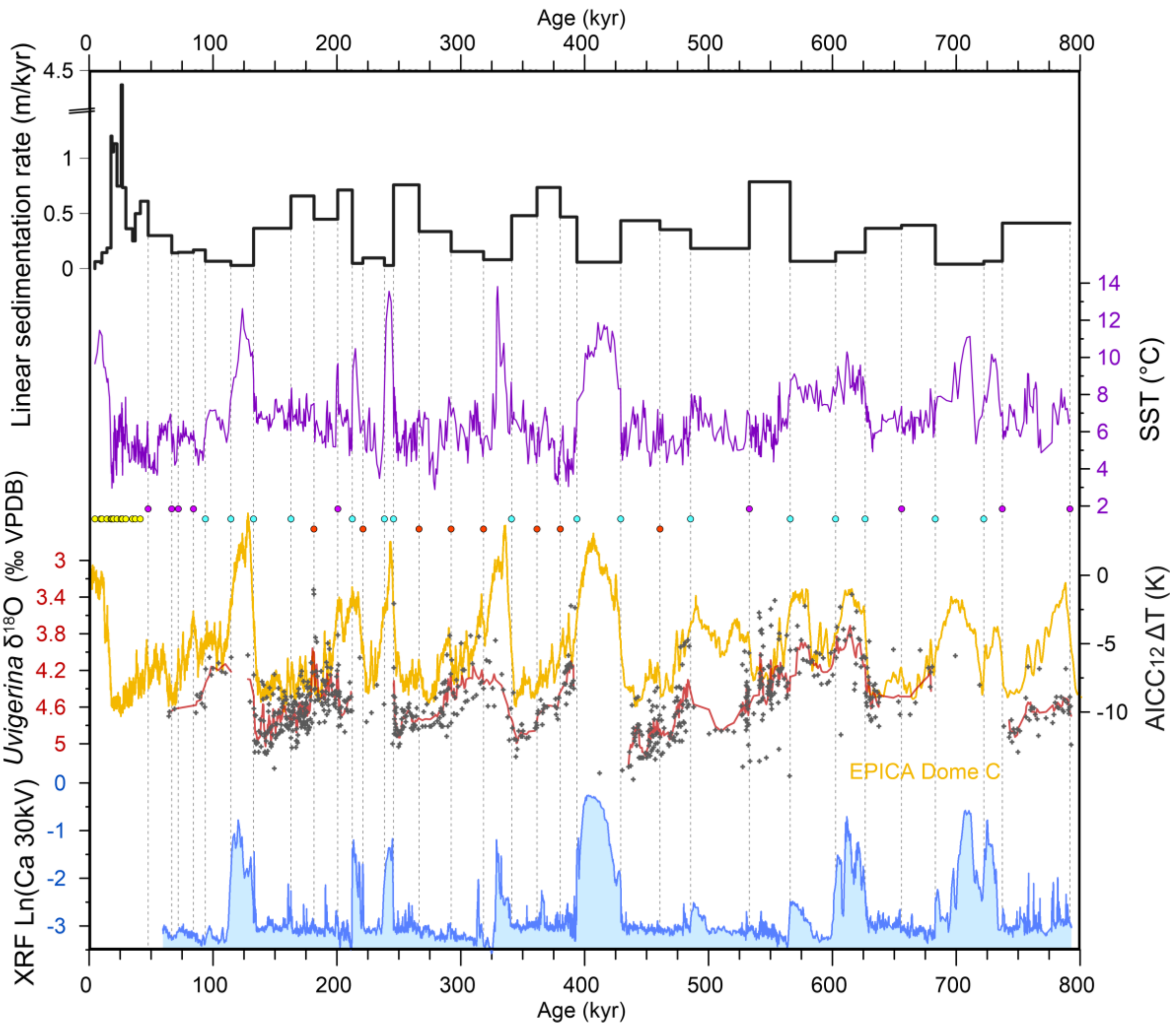

Supplementary figure 3. Chronology and constraint for the age model construction for Site U1542. From top to bottom, resulting linear sedimentation rate (LSR), sea surface temperature (SST) reconstruction from Site U1542, $\delta^{18}O$ record from *Uvigerina* (benthic) foraminifera isotopes, Antarctic ice core EPICA Dome C temperature record[7] on the AICC2012 age model[8] and XRF-derived ln(Ca) from Site U1542. The target used was the Antarctic ice core temperature record. The orbital correlation at the glacial/interglacial boundary was performed using ln(Ca) (blue dots). As several transitions were not well marked in the ln(Ca) record, alkenones-derived SST (purple dots) was used. Subsequently, the benthic $\delta^{18}O$ record was used to align our records with the Antarctic isotope maxima (pink dots). The first 60 kyr, based on MD07-3128 follows the latest age model Anderson et al.[9]. All tuning was performed using AnalySeries[6].

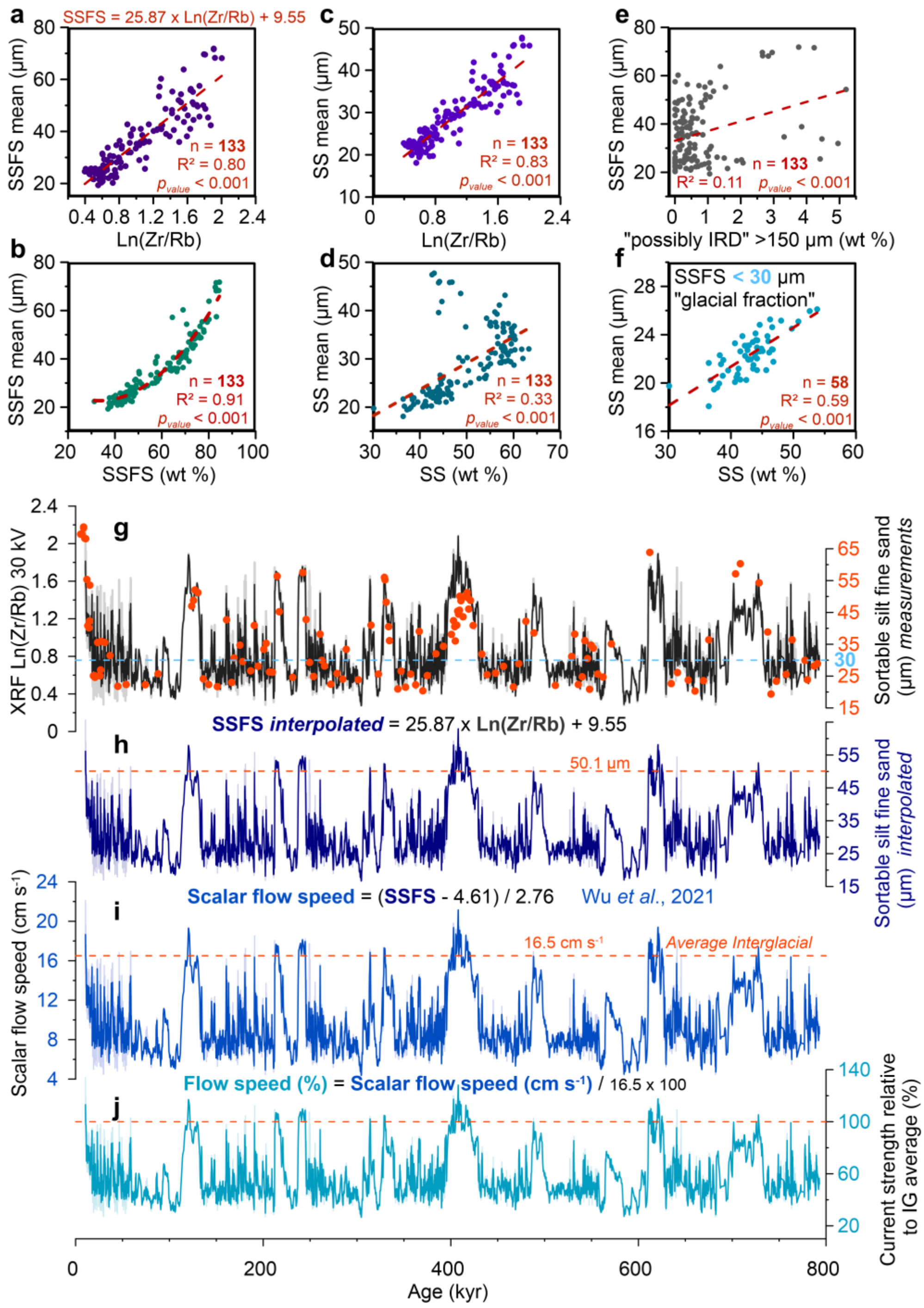

Supplementary figure 4. Path of different steps to reconstruct the Cape Horn Current strength proxy relative to the average interglacial mean. (**a**) Linear regression between mean sortable silts plus fine sand fraction (SSFS) and XRF-derived ln(Zr/Rb). (**b**) Polynomial regression between mean SSFS and the weight percentage of SSFS suggesting of a current controlled deposit. (**c**) Mean sortable silts fraction (SS) and XRF-derived ln(Zr/Rb). (**d**) Linear regression between mean SS and the weight percentage of SS. (**e**) Mean SSFS are independent of the coarser (>150 µm) "possible IRD" fraction. (**f**) The SSFS < 30 µm deposited during colder periods, i.e. "glacial fraction" present relatively good correlation between mean SS and percentage of SS, suggesting of moderate influence of terrestrial source during glacial periods. (**g**) XRF-derived ln(Zr/Rb) (black) with the 133 grain-size measurements along the record (orange dots). (**h**) SSFS interpolated using the linear regression SSFS = 25.87 x ln(Zr/Rb) + 9.55. (**i**) Scalar flow speed derived from SSFS using Wu calibration[10]. (**j**) Scalar flow speed related to the full interglacial mean value expressed as a percentage.

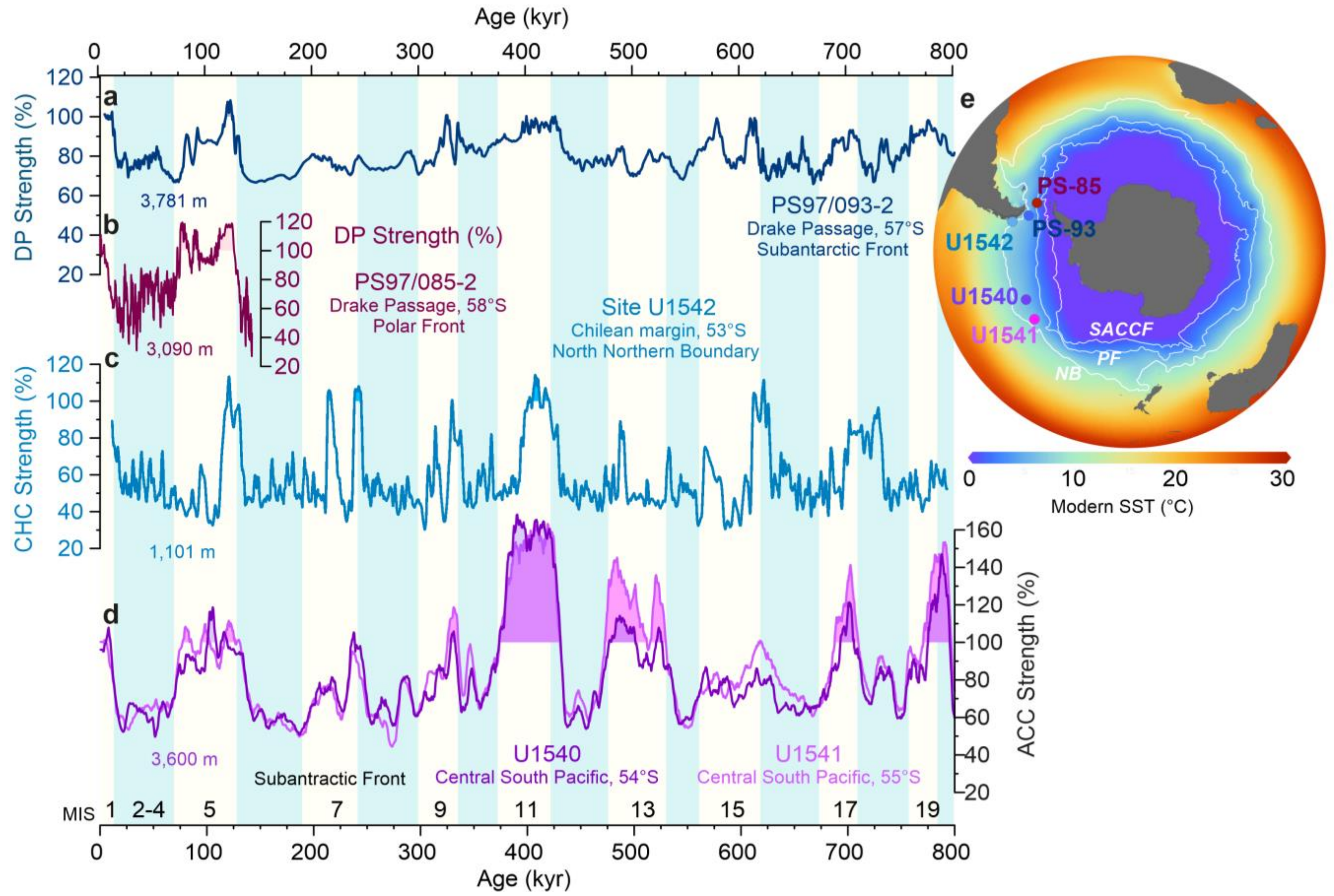

Supplementary figure 5. **Pacific Antarctic Circumpolar Current strength changes over the past 800 kyr in the subantarctic Pacific**. (**a**) Reconstructed relative current strength variations (compared with Holocene mean values) in the Drake Passage (DP)[11,12], (**b**) Reconstructed high resolution relative current strength variations in the DP near the Polar Front[10]. (**c**) Reconstructed relative current strength variations on the Chilean margin at the Cape Horn Current (CHC; this study). (**d**) Reconstructed relative current strength variations in the central subantarctic Pacific in the core of the Antarctic Circumpolar Current (ACC)[12]. Latitude, location related to oceanic fronts, and water depth are indicated by identical color to the record. The map (**e**) indicates different locations of plotted records. Modern sea surface temperature (SST) is from the World Ocean Atlas (based on 2005-2017 average observations)[13], and the map was created using Ocean Data View[14]. Lines represent altimetry-derived ACC fronts[15]; NB = Northern Boundary, PF = Polar Front, SACCF = Southern ACC Front.

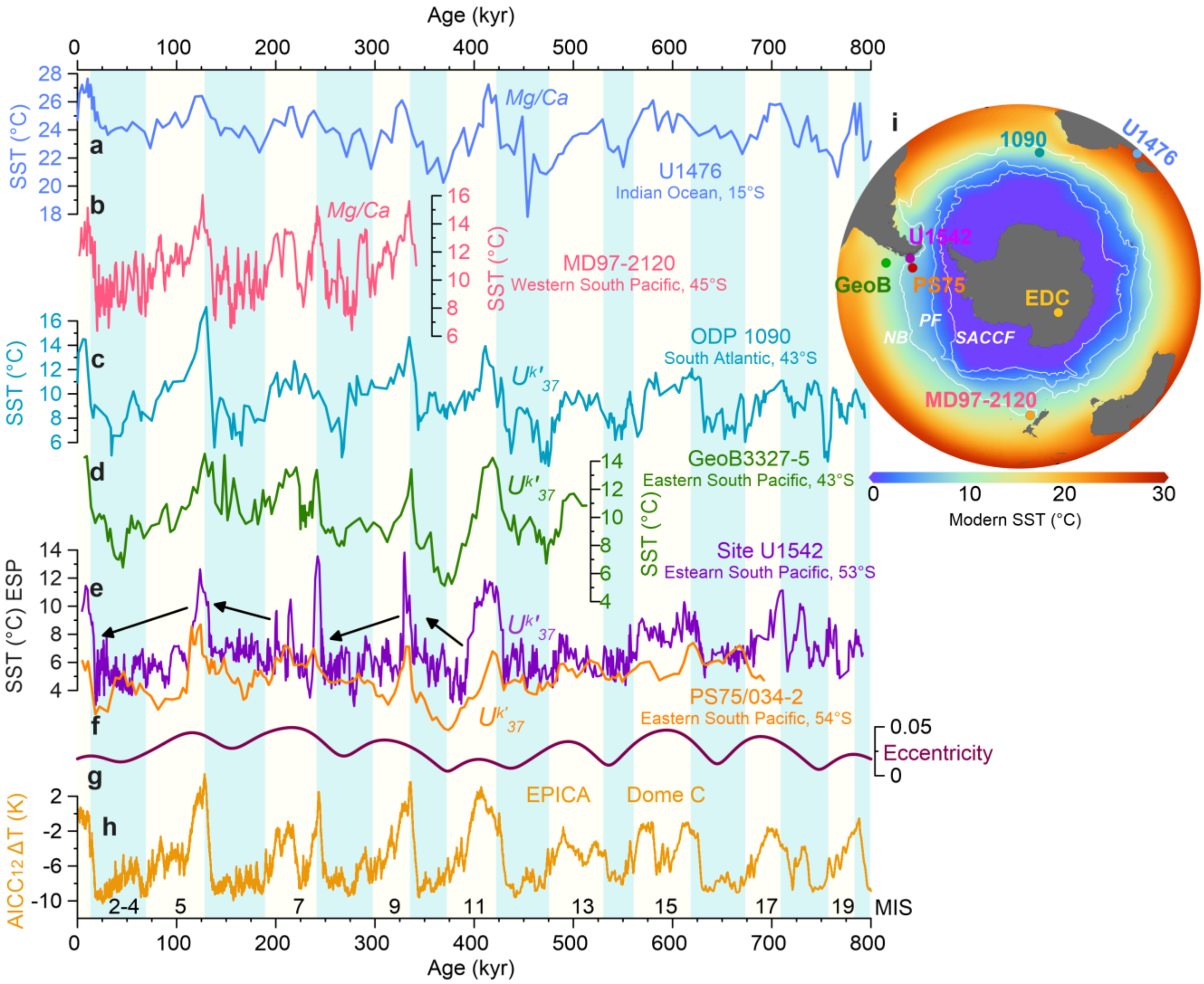

Supplementary figure 6. Sea surface temperature reconstructions from the Southern Hemisphere. (**a**) Mg/Ca-derived sea surface temperature (SST) from the west Indian Ocean. (**b**) Mg/Ca-derived SST from the western South Pacific[16]. (**c**) alkenone-derived SST from the Subantarctic Atlantic[17]. (**d**), (**e**, this study) and (**f**)[18] alkenone-derived SST from the eastern South Pacific. (**h**) Antarctic atmospheric temperature of ice core record[7] on the AICC2012 age model[8]. (**g**) Mid-latitude SST records largely follow the 100 kyr eccentricity component. (**i**) The map indicates different locations of temperature records. Modern SST is from the World Ocean Atlas (based on 2005-2017 average observations)[13], and the map was created using Ocean Data View[14]. Lines represent altimetry-derived ACC fronts[15]; NB = Northern Boundary, PF = Polar Front, SACCF = Southern ACC Front.

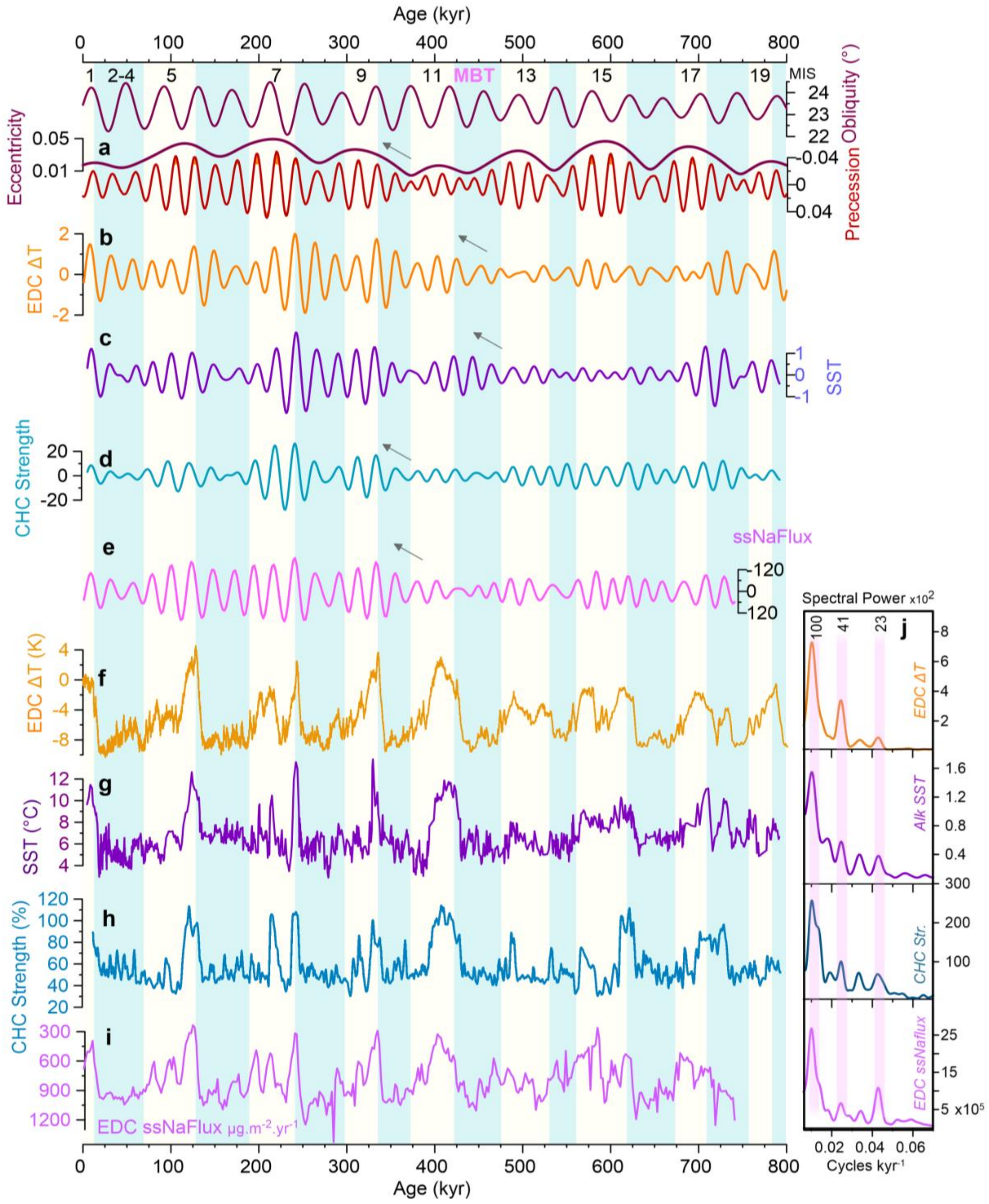

Supplementary figure 7. Orbital changes at Site U1542. (**a**) Orbital parameters, obliquity, eccentricity and precession[19]. (**b**) 23-kyr component of Antarctic atmospheric temperature ice core record[7]. (**c**) 23-kyr component of sea surface temperature (SST). (**d**) 23-kyr component of Cape Horn Current (CHC) strength records from Site U1542. (**c**) 23-kyr component of the sea salt sodium (ssNa+) flux record from EDC, a proxy for sea ice extent and atmosphere changes[20]. (**f** to **i**) Their respective original data. (**j**) Their respective spectral analyses.

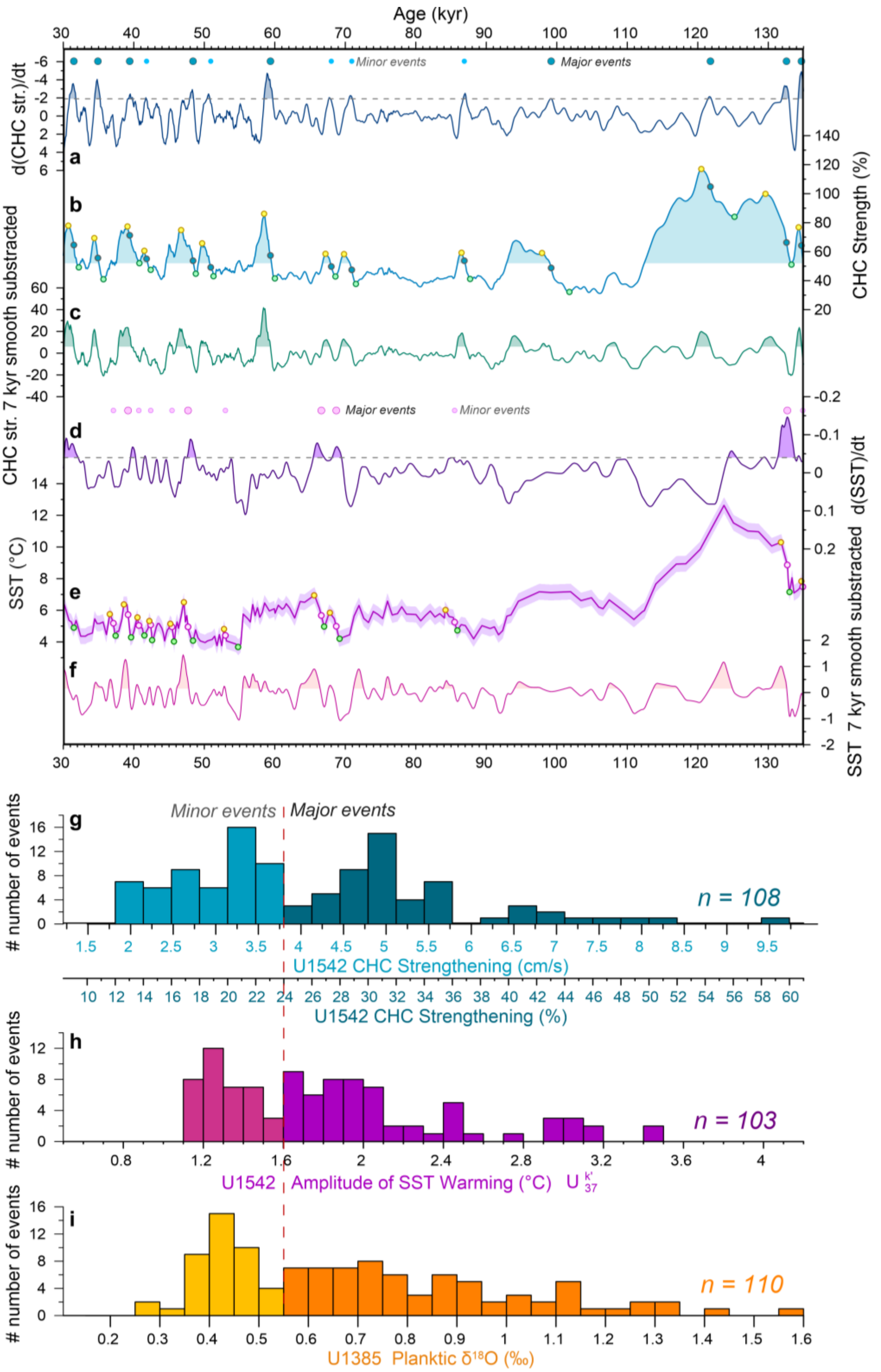

Supplementary figure 8. Visualization of different steps to identify millennial-scale climatic events. The selection threshold was applied to the minima of the first differential (**a** and **d**). The resulting curve is similar to the millennial component (**c** and **f**). "Filtered", i.e., removal of orbital-timescale variability of the original signal (**b** and **e**) is achieved by subtracting a 7 kyr smooth. The green dots indicate the initiation of the event, the corresponding color dots indicate the highest rate of change (maxima of the differential), and the light-yellow dots indicate the termination of the event. (**g**) Sorting of Cape Horn Current (CHC) Strength events recorded at Site U1542. (**h**) Sorting of sea surface temperature (SST) events recorded at Site U1542. (**i**) Sorting of stadial events recorded at Site U1385[21], according to their respective amplitude. The results indicate two distributions, used to separate major with minor events.

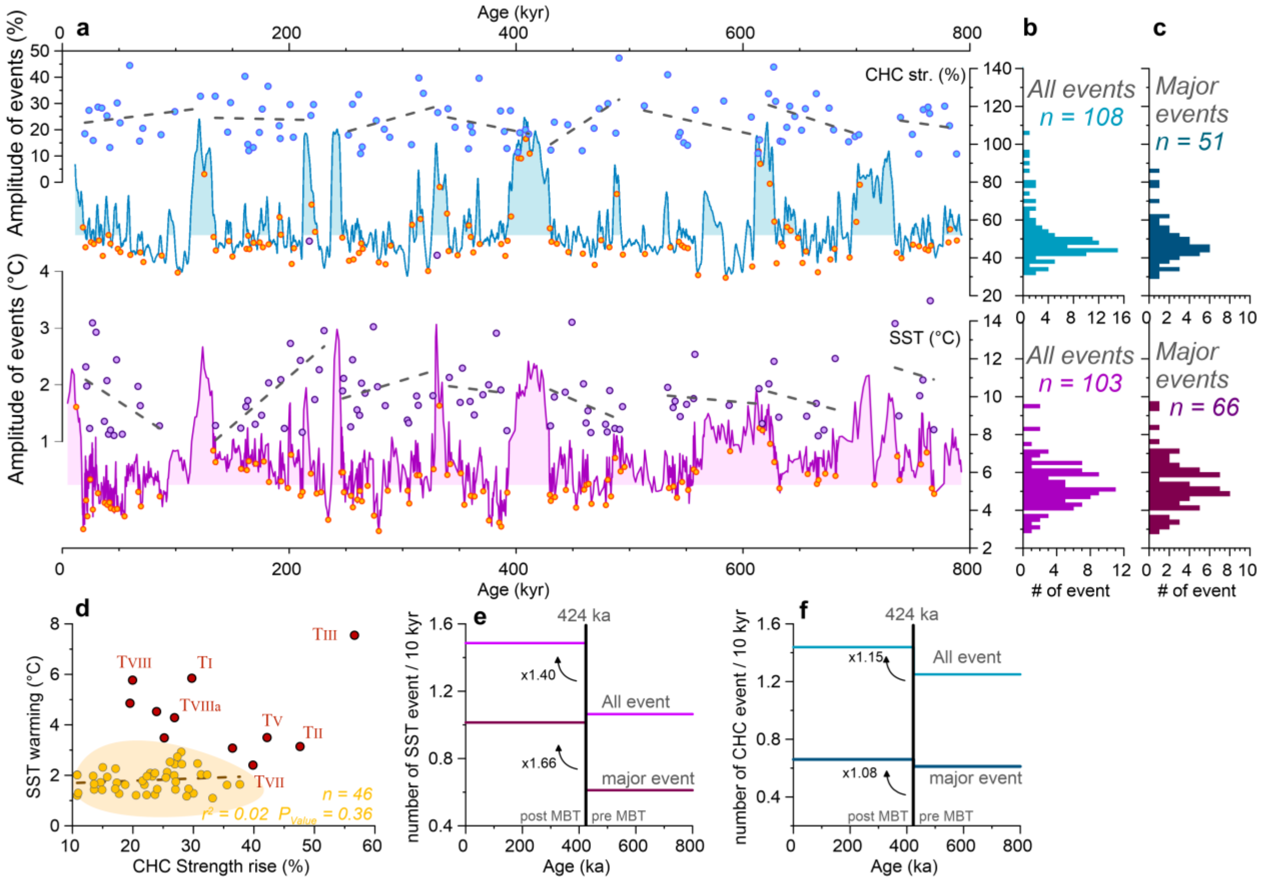

Supplementary figure 9. Millennial-scale events at Site U1542. (**a**) Amplitude of Cape Horn Current (CHC) strengthening and SST warming events recorded at Site U1542. (**b**) Distribution of millennial-scale events recorded at Site U1542 according to the condition at the initiation. (**c**) Same but for the major events only. (**d**) Relation between SST warming millennial-scale events occurring synchronously with CHC strengthening millennial-scale events in less than 1 kyr (yellow dots). Red dots indicate SST warming and CHC strengthening associated with glacial terminations. (**e**) and (**f**) The number of events / 10 kyr before and after the Mid Brunhes Transition (MBT) of SST and of CHC, respectively.

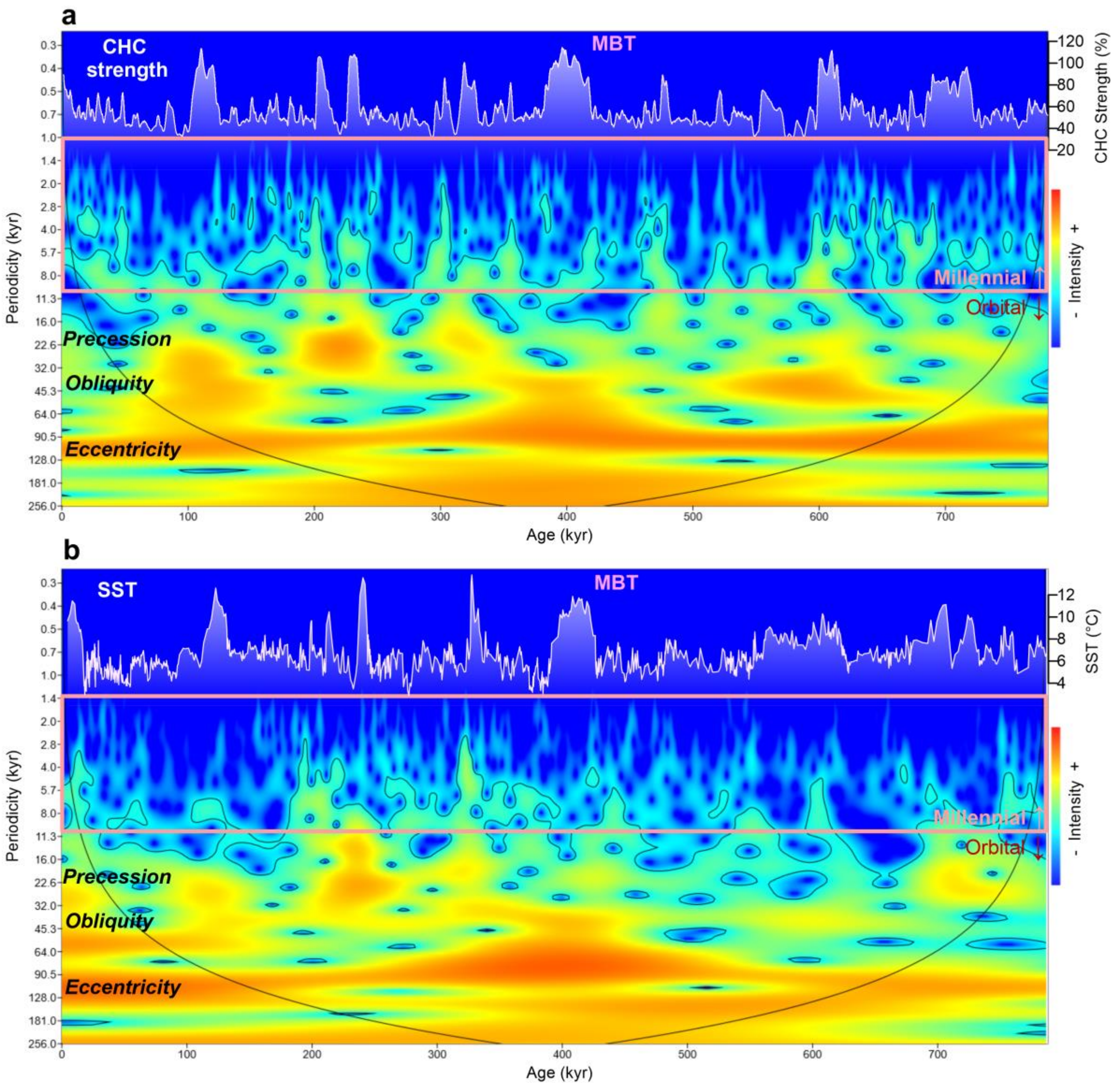

Supplementary figure 10. Continuous wavelet transform (**a**) Continuous wavelet transform of the Cape Horn Current (CHC) strength, (**b**) Continuous wavelet transform of sea surface temperature (SST) at Site U1542. The continuous wavelet transform was computed using the Morlet basis function in PAST[22]. The black line underlines the significance level (p=0.05) and the parabolic line is the cone of influence. MBT = Mid-Bruhnes Transition. Orbital climate windows can be defined as having a periodicity between 10^4 and 10^5 years. Millennial climate windows can be defined as having a periodicity between 10^3 and 10^4 years. Millennial-scale climate variability shows stronger periodicity between ~5 and ~8 kyr. The millennial-scale SST variability appears more intense from 400 ka to 180 ka, coinciding with higher amplitude during glacial cycles 3 and 4 (Fig. 4a).

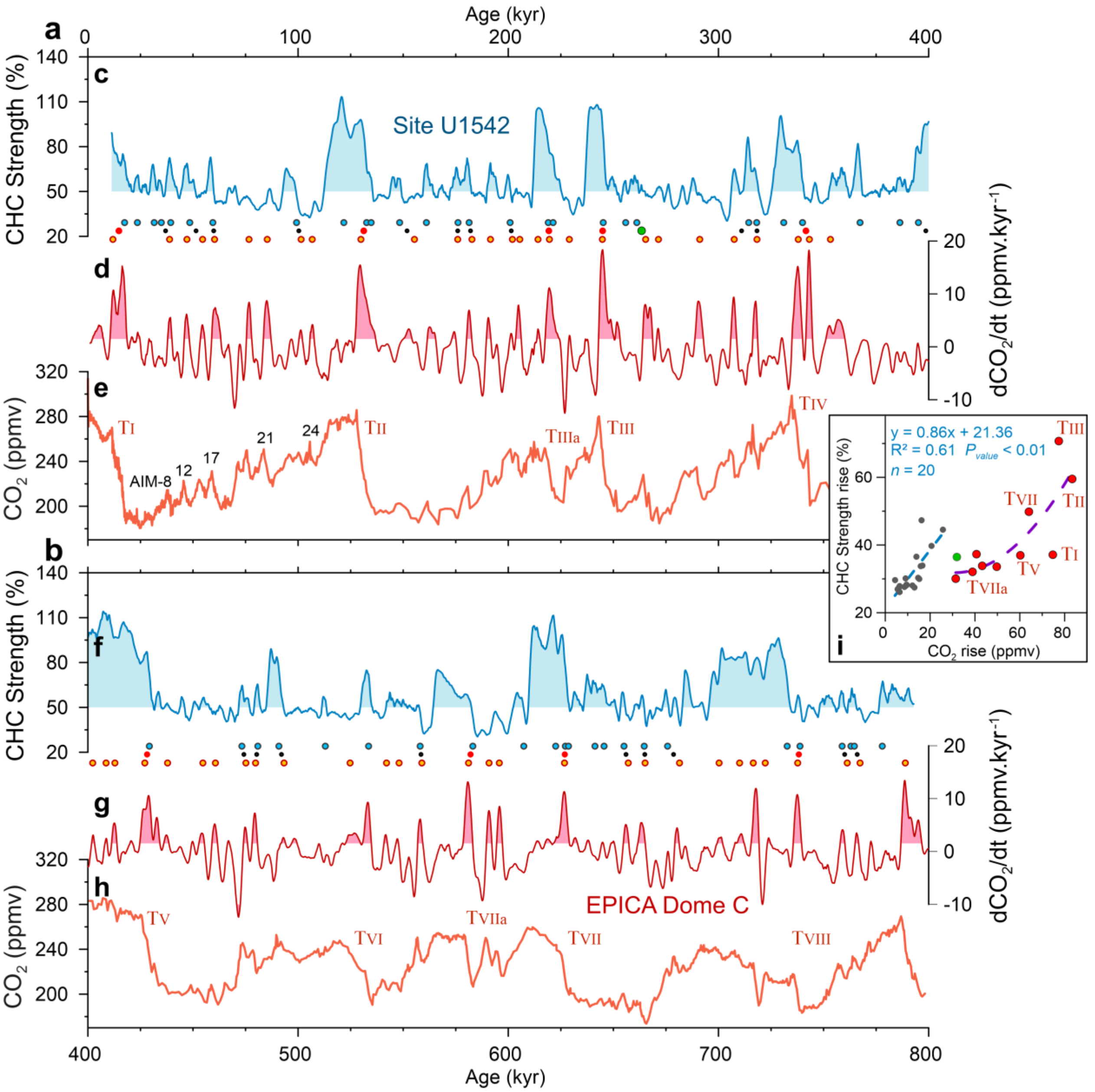

Supplementary figure 11. Rate of atmospheric CO_2 changes and Antarctic Circumpolar Current during the Pleistocene. (**a**) Climate reconstruction from 0 to 400 kyr. (**b**) Climate reconstruction from 400 to 800 kyr. (**c, f**) CHC strength from Site U1542. (**d, g**) Rate of atmospheric CO_2 changes (dCO_2/dt) from[23] based on (**e, h**). (**e, h**) Atmospheric CO_2 concentrations from the EPICA Dome C ice core[24]. (**i**) Relation between magnitude (%) of the CHC/ACC events with rise of CO_2 (ppmv) observed. Blue dots indicate major CHC strengthening events, yellow dots indicate CO_2 increase events, and black dots indicate association (n = 31) between both events in less than 7 kyr. We associated 31 major CHC/ACC strengthening events with CO_2 rise in less than 7 kyr over the past 790 kyr. Among these 31 events, 10 (red dots) events are associated with terminations and the 264-ka event (green dot) are removed from the linear correlation due to its similitude with glacial terminations.

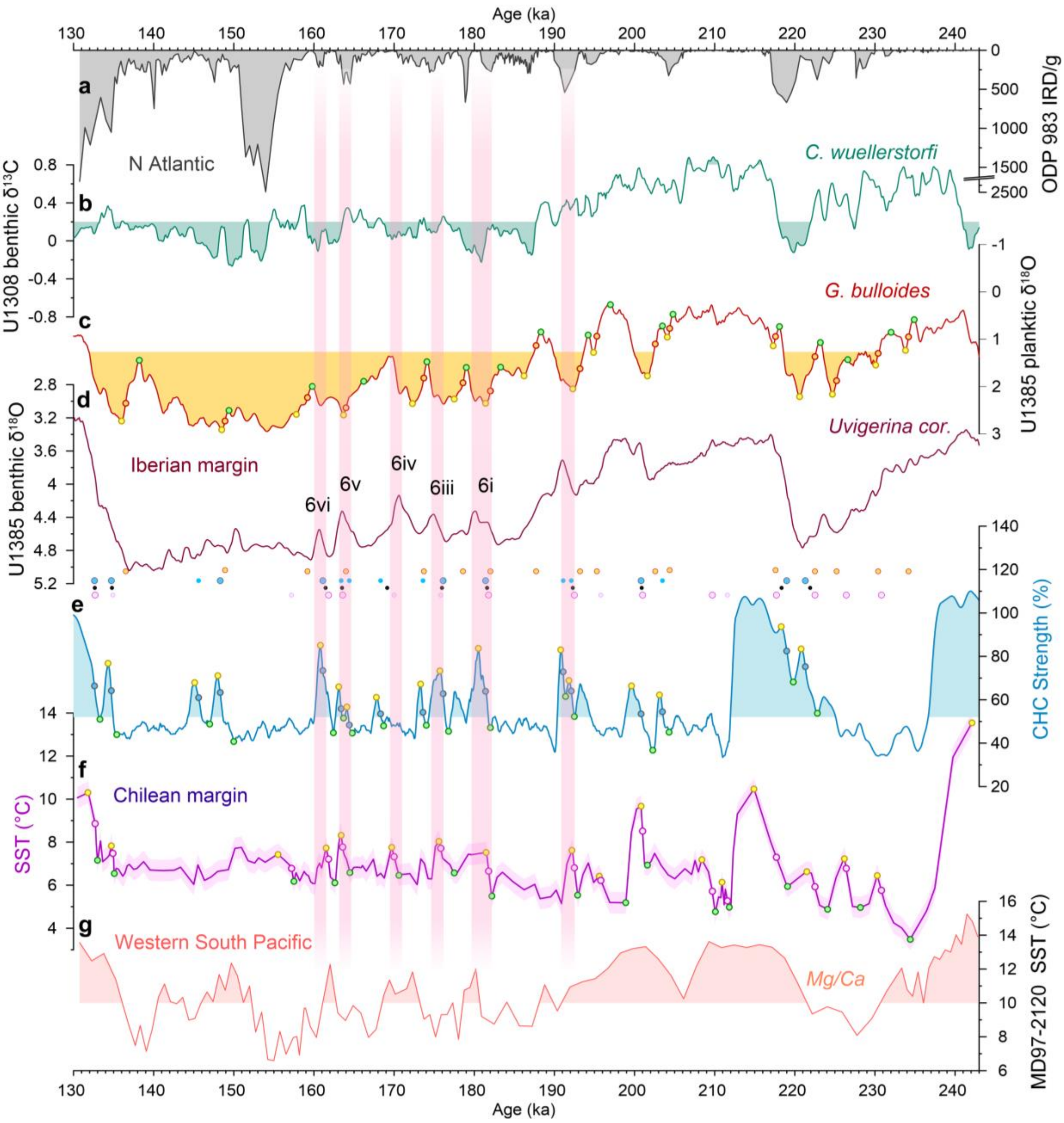

Supplementary figure 12. Zoom on the penultimate glacial cycle (130-234 ka). (**a**) Ice-rafted debris at ODP Site 983[23]. (**b**) Benthic δ^{13}C from Site U1308 indicating mixing ratio between northern and southern sourced waters[25]. (**c**) Planktic δ^{18}O from Site U1385[21] taken as proxy for sea surface temperature (SST) changes. (**d**) Benthic δ^{18}O from Site U1385[21]. (**e**) Cape Horn Current (CHC) strength from Site U1542 (this study. (**f**) SST with uncertainty envelope (0.5°C; see methods) from Site U1542 (this study). (**g**) Mg/Ca-derived SST from the Southwest Pacific[16]. The green dots indicate the initiation of the event, the corresponding color dots indicate the highest rate of change (maxima of the differential), and the light-yellow dots indicate the termination of the event. The nomenclature of events follow Margari et al.,[26]. Vertical pink bars indicate major events. In this glacial cycle, there are 17 NH cooling events (average amplitude of 0.8 ‰), 17 SH warming events (average amplitude of 2.05°C) and 17 CHC strengthening events (average amplitude of 24.8 %)

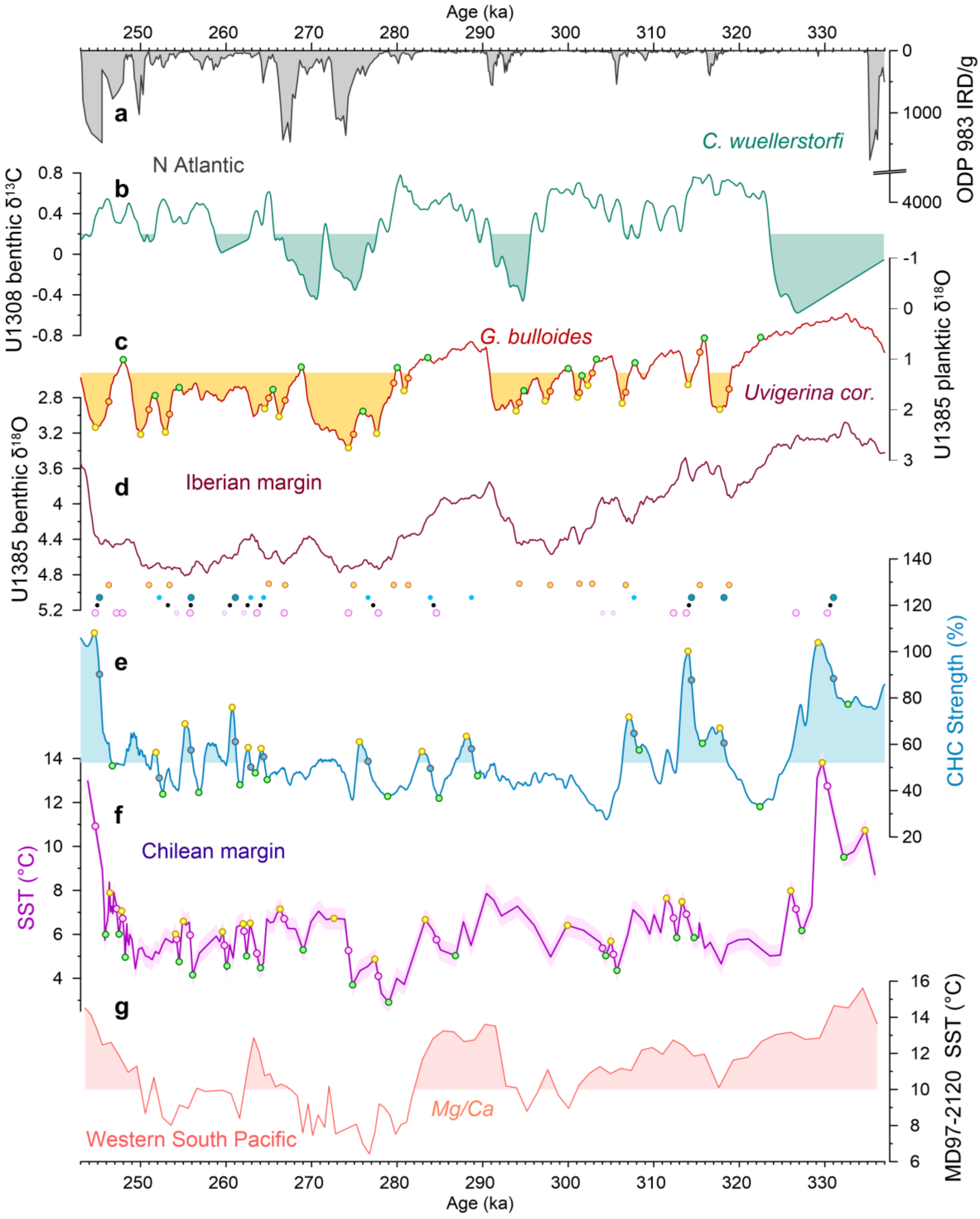

Supplementary figure 12b. Zoom on the glacial cycle number 3 (234-337 ka). In this glacial cycle, there are 15 NH cooling events (average amplitude of 0.81 ‰), 18 SH warming events (average amplitude of 2.28°C) and 13 CHC strengthening events (average amplitude of 26 %).

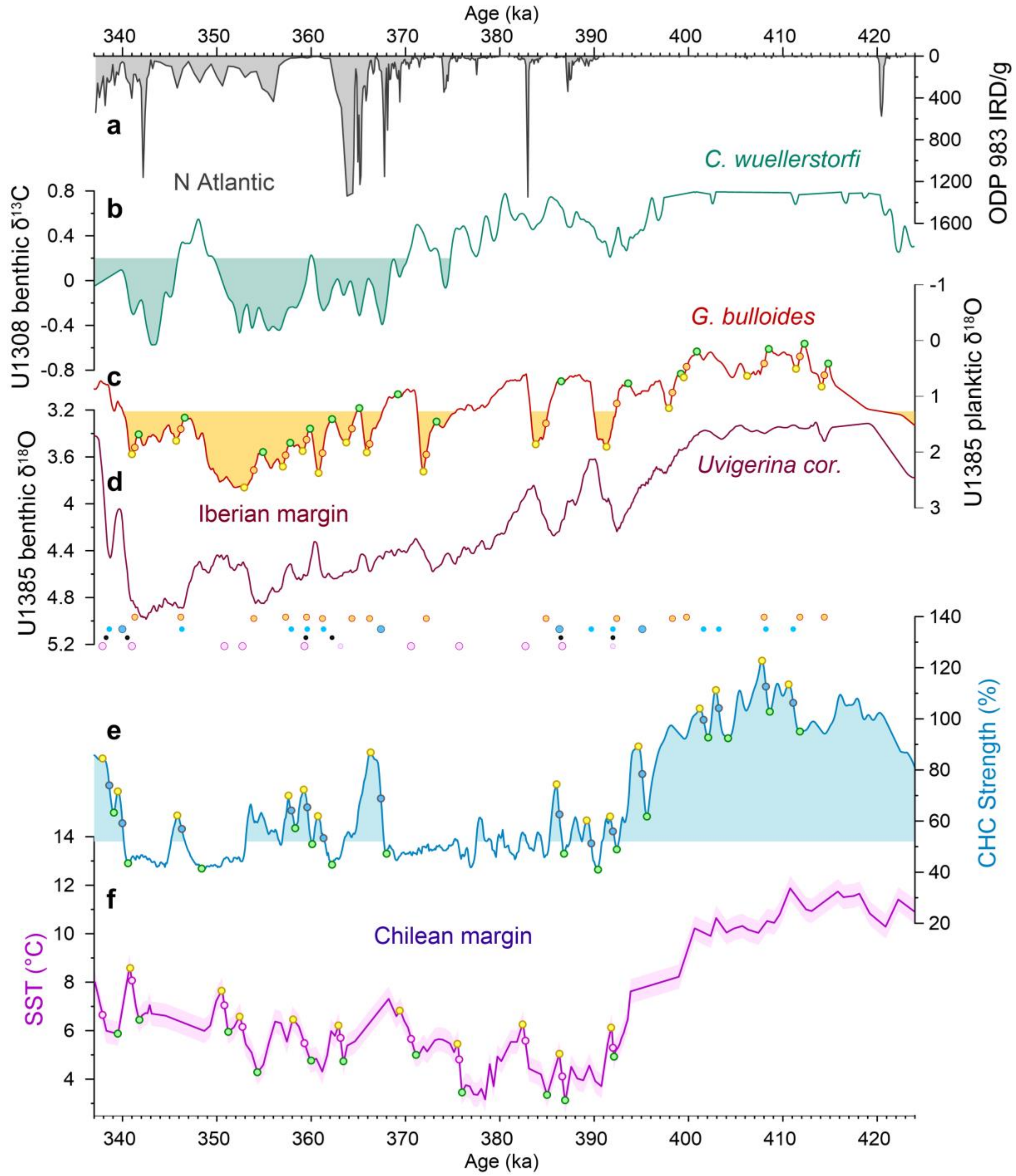

Supplementary figure 12c. Zoom on the glacial cycle number 4 (337-424 ka). In this glacial cycle, there are 16 NH cooling events (average amplitude of 0.65 ‰), 11 SH warming events (average amplitude of 2.17°C) and 15 CHC strengthening events (average amplitude of 21.2 %).

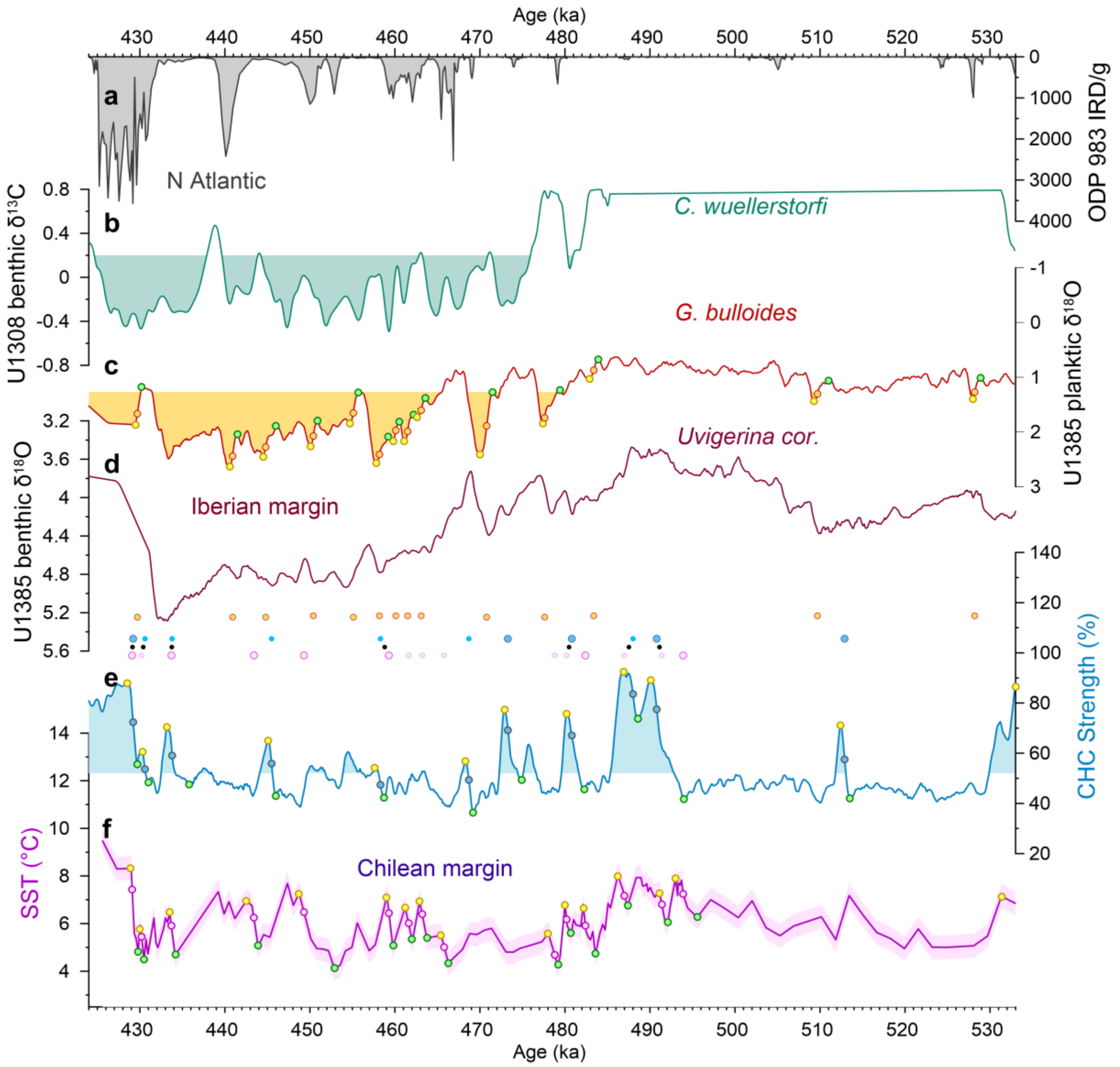

Supplementary figure 12d. Zoom on the glacial cycle number 5. **(424-533 ka)**. In this glacial cycle, there are 14 NH cooling events (average amplitude of 0.53 ‰), 15 SH warming events (average amplitude of 1.73°C) and 11 CHC strengthening events (average amplitude of 25 %).

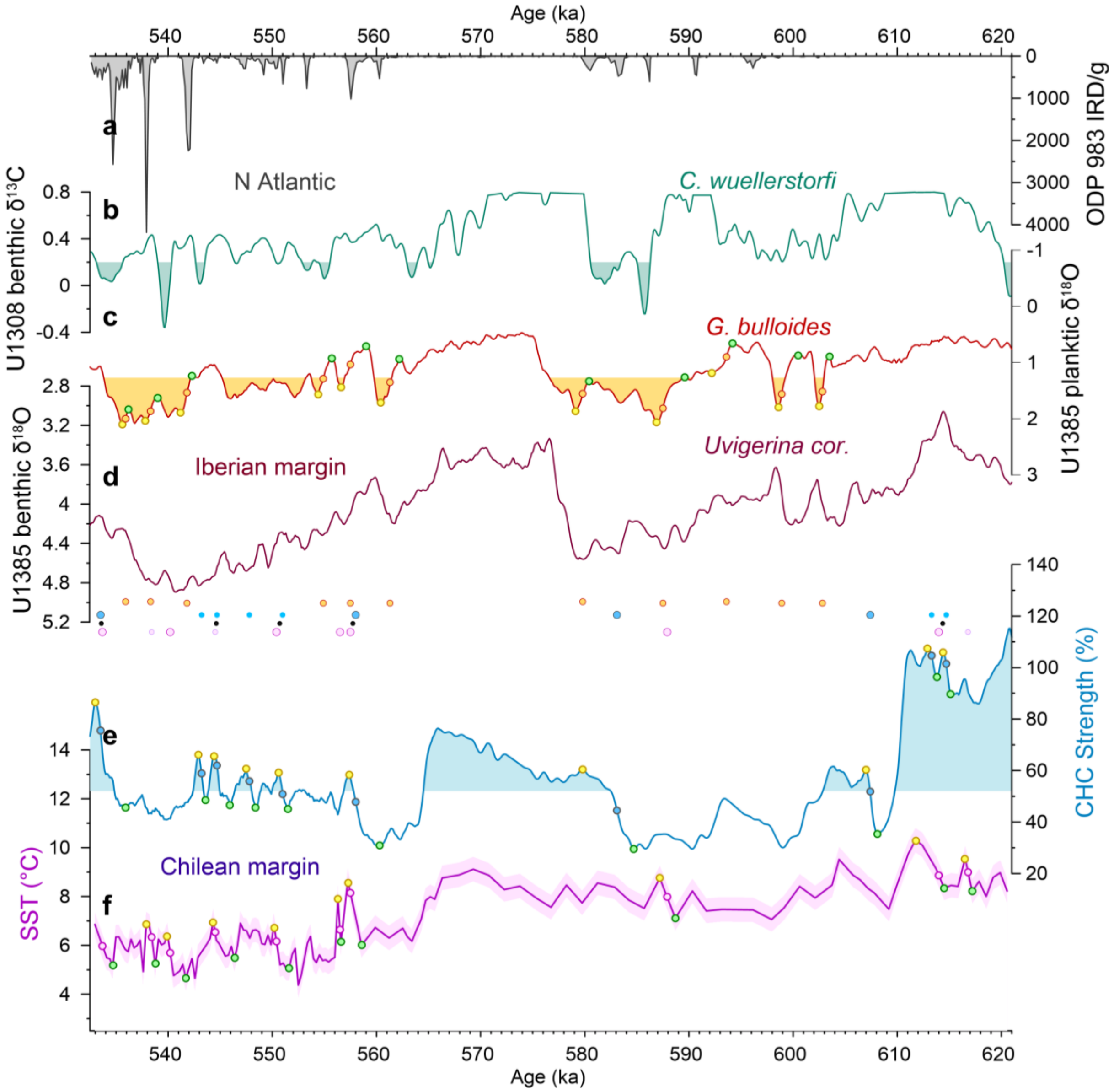

Supplementary figure 12e. Zoom on the glacial cycle number 6 (533-621 ka). In this glacial cycle, there are 11 NH cooling events (average amplitude of 0.65 ‰), 10 SH warming events (average amplitude of 1.75°C) and 10 CHC strengthening events (average amplitude of 21.7 %).

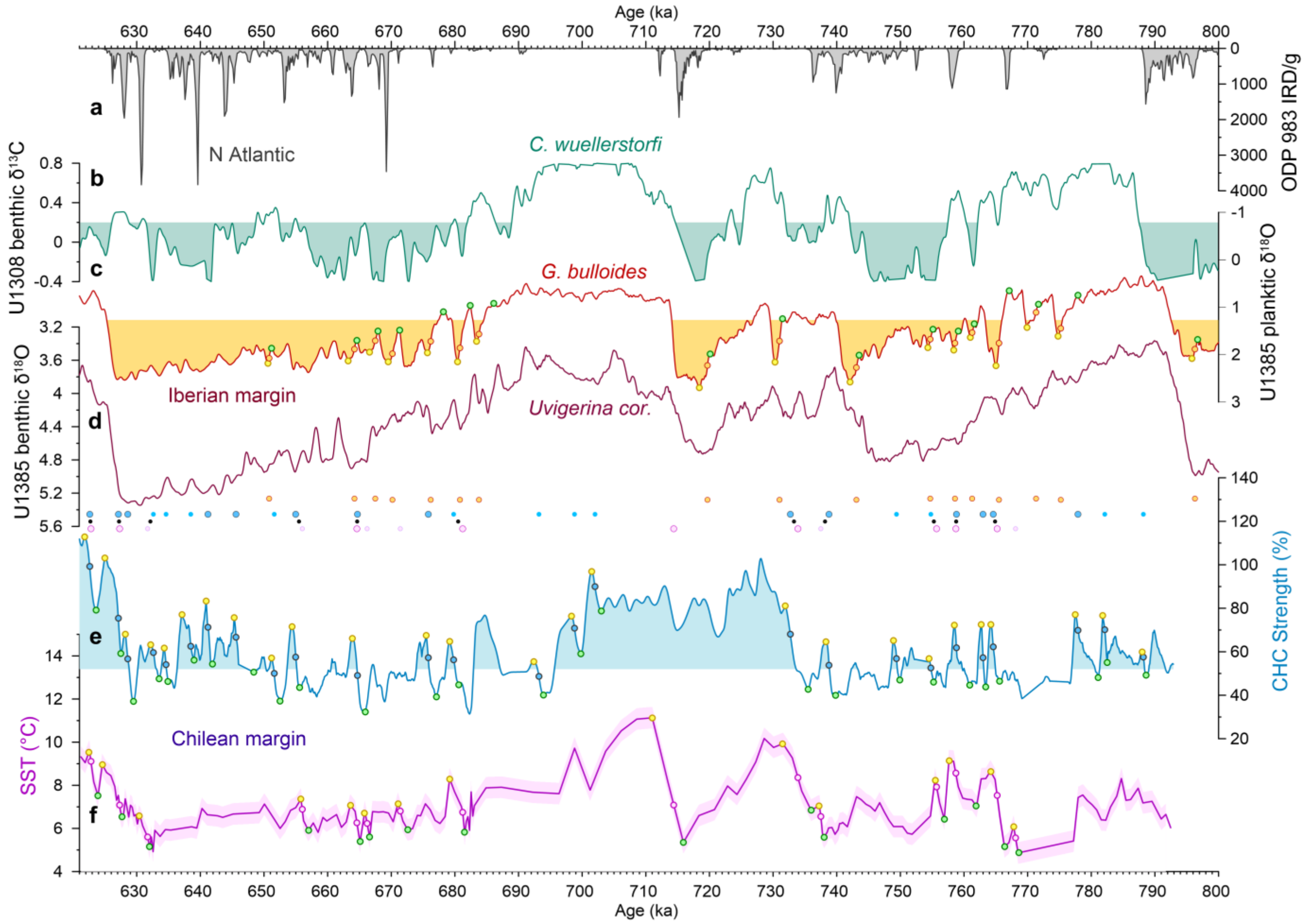

Supplementary figure 12f. Zoom on the glacial cycle number 7 (621-800 ka). Here, the glacial cycles MIS 16-17 and MIS 18-19 are merged as the MIS 19/20 boundary is the bottom of the record and not well constrained. In these two glacial cycles, there are 17 NH cooling events (average amplitude of 0.67 ‰), 15 SH warming events (average amplitude of 2.18°C) and 26 CHC strengthening events (average amplitude of 24.3 %).

Supplementary References

1. Chaigneau, A. & Pizarro, O. Surface circulation and fronts of the South Pacific Ocean, east of 120°W. *Geophys. Res. Lett.* **32**, (2005).
2. Strub, P. T., James, C., Montecino, V., Rutllant, J. A. & Blanco, J. L. Ocean circulation along the southern Chile transition region (38°–46°S): Mean, seasonal and interannual variability, with a focus on 2014–2016. *Prog. Oceanogr.* **172**, 159–198 (2019).
3. Zheng, Q., Bingham, R. & Andrews, O. Using Sea Level to Determine the Strength, Structure and Variability of the Cape Horn Current. *Geophys. Res. Lett.* **50**, e2023GL105033 (2023).
4. Saldías, G. S. *et al.* Satellite-Derived Variability of Sea Surface Salinity and Geostrophic Currents off Western Patagonia. *Remote Sens.* **16**, 1482 (2024).
5. Lamy, F. *et al.* Glacial reduction and millennial-scale variations in Drake Passage throughflow. *Proc. Natl. Acad. Sci.* **112**, 13496–13501 (2015).
6. Paillard, D., Labeyrie, L. & Yiou, P. Macintosh Program performs time-series analysis. *EOS Trans.* **77**, 379–379 (1996).
7. Jouzel, J. *et al.* Orbital and Millennial Antarctic Climate Variability over the Past 800,000 Years. *Science* **317**, 793–796 (2007).
8. Bazin, L. *et al.* An optimized multi-proxy, multi-site Antarctic ice and gas orbital chronology (AICC2012): 120–800 ka. *Clim. Past* **9**, 1715–1731 (2013).
9. Anderson, H. J., Pedro, J. B., Bostock, H. C., Chase, Z. & Noble, T. L. Compiled Southern Ocean sea surface temperatures correlate with Antarctic Isotope Maxima. *Quat. Sci. Rev.* **255**, 106821 (2021).
10. Wu, S. *et al.* Orbital- and millennial-scale Antarctic Circumpolar Current variability in Drake Passage over the past 140,000 years. *Nat. Commun.* **12**, 3948 (2021).
11. Toyos, M. H. *et al.* Antarctic Circumpolar Current Dynamics at the Pacific Entrance to the Drake Passage Over the Past 1.3 Million Years. *Paleoceanogr. Paleoclimatology* **35**, (2020).
12. Lamy, F. *et al.* Five million years of Antarctic Circumpolar Current strength variability. *Nature* **627**, 789–796 (2024).
13. Locarnini, M. *et al.* World Ocean Atlas 2018, Volume 1: Temperature. (2018).
14. Schlitzer, R. Ocean Data View. (2025).
15. Park, Y.-H. *et al.* Observations of the Antarctic Circumpolar Current Over the Udintsev Fracture Zone, the Narrowest Choke Point in the Southern Ocean. *J. Geophys. Res. Oceans* **124**, 4511–4528 (2019).

16. Pahnke, K., Zahn, R., Elderfield, H. & Schulz, M. 340,000-Year Centennial-Scale Marine Record of Southern Hemisphere Climatic Oscillation. *Science* **301**, 948–952 (2003).

17. Martínez-Garcia, A., Rosell-Melé, A., McClymont, E. L., Gersonde, R. & Haug, G. H. Subpolar Link to the Emergence of the Modern Equatorial Pacific Cold Tongue. *Science* **328**, 1550–1553 (2010).

18. Ho, S. L. *et al.* Sea surface temperature variability in the Pacific sector of the Southern Ocean over the past 700 kyr. *Paleoceanography* **27**, (2012).

19. Berger, A., Loutre, M. F. & Laskar, J. Stability of the Astronomical Frequencies Over the Earth's History for Paleoclimate Studies. *Science* **255**, 560–566 (1992).

20. Wolff, E. W. *et al.* Southern Ocean sea-ice extent, productivity and iron flux over the past eight glacial cycles. *Nature* **440**, 491–496 (2006).

21. Hodell, D. A. *et al.* A 1.5-million-year record of orbital and millennial climate variability in the North Atlantic. *Clim. Past* **19**, 607–636 (2023).

22. Hammer, O. & Harper, D. A. Past: paleontological statistics software package for educaton and data anlysis. *Palaeontol. Electron.* **4**, 1 (2001).

23. Barker, S. *et al.* Early Interglacial Legacy of Deglacial Climate Instability. *Paleoceanogr. Paleoclimatology* **34**, 1455–1475 (2019).

24. Bereiter, B. *et al.* Revision of the EPICA Dome C CO2 record from 800 to 600 kyr before present. *Geophys. Res. Lett.* **42**, 542–549 (2015).

25. Hodell, D. A., Channell, J. E. T., Curtis, J. H., Romero, O. E. & Röhl, U. Onset of "Hudson Strait" Heinrich events in the eastern North Atlantic at the end of the middle Pleistocene transition (~640 ka)? *Paleoceanography* **23**, (2008).

26. Margari, V. *et al.* The nature of millennial-scale climate variability during the past two glacial periods. *Nat. Geosci.* **3**, 127–131 (2010).
